# Invariant and suboptimal trajectories of self-regulated learning during secondary school: implications focused on quality in higher education

**DOI:** 10.3389/fpsyg.2023.1235846

**Published:** 2023-07-19

**Authors:** Fabiola Sáez-Delgado, Javier Mella-Norambuena, Yaranay López-Angulo, Yenniffer Sáez, Verónica León-Ron

**Affiliations:** ^1^Departamento Fundamentos de la Pedagogía, Facultad de Educación, Centro de Investigación en Educación y Desarrollo (CIEDE-UCSC), Universidad Católica de la Santísima Concepción, Concepción, Chile; ^2^Departamento de Ciencias, Universidad Técnica Federico Santa María, Concepción, Chile; ^3^Departamento de Psicología, Facultad de Ciencias Sociales, Universidad de Concepción, Concepción, Chile; ^4^Programa de Magíster en Ciencias de la Educación, Universidad Católica de la Santísima Concepción, Concepción, Chile; ^5^Facultad de Educación, Ciencia y Tecnología, Universidad Técnica del Norte, Ibarra, Ecuador

**Keywords:** self-regulated learning, secondary school, transition to higher education, quality of education, longitudinal study

## Abstract

It has been shown that self-regulation of learning is a key variable for an adequate transition and adjustment from secondary school to tertiary education, and it is also associated with successful academic results; therefore, it is relevant to analyze its levels of development in the pre-university stage. The aim of this research was to evaluate the trajectories of self-regulation of learning in secondary school students. The method considered a longitudinal design and included a sample of 403 students from 9^th^ to 12^th^ grade in Chile. An instrument with adequate psychometric properties was used to measure the learning self-regulation process (disposition, performance and self-evaluation phases). The results showed that self-regulation is at suboptimal levels in its different phases (*M* = 4.25 to *M* = 4.71). Linear mixed models showed: a significant effect of sex on the disposition variable in favor of females; and that the phases of disposition, performance and self-evaluation do not change over time. It is concluded that, if self-regulation of learning is not specifically trained, it does not increase during secondary school. The findings are discussed considering the possible practical implications for educational policies, research, timely intervention and impact on the quality of school and university education.

## 1. Introduction

### 1.1. Changes in society and new educational requirements in higher education

In the last decades, important changes in the development of competency-based educational programs can be identified, also, to considerable progress in information and communication technologies, and accelerated globalization, implying multiple challenges in the world of education (Espada et al., 2020). In this context, universities around the world have been striving to move from a solely knowledge-centered approach to a broader competency-based approach in university curricula to drive improvement in the quality of teaching-learning processes (Hensley et al., 2021). In other words, curricula should incorporate, not only disciplinary content, but also those skills that will enable students to become future independent and productive individuals in their society and to contribute to the progress of their country (Mustapha et al., 2023). Therefore, higher education has the responsibility to proceed with high efficiency in the academic and professional preparation of young people entering university. It is required to train the student body for in-depth learning, and critical thinking, enabling them to adjust and respond to the changes that have occurred in today’s society, and take responsibility for their learning and future professional work (Šteh and Šarić, 2020).

To achieve these objectives, one of the main generic competencies of Higher Education that needs to be developed is lifelong learning (“Learning to Learn”), whose key component or cornerstone is the ability to self-regulate learning (SRL) (Lluch and Cano, 2023). Therefore, at present, in order to achieve teaching-learning processes that pursue as an ultimate goal the improvement of the quality of Higher Education, it is necessary to consider solid training in this competence, i.e., as a learning outcome to be achieved (Anthonysamy et al., 2020b). That is, SRL should be pursued intentionally and systematically (Lluch and Cano, 2023).

### 1.2. Model and conceptualization of self-regulation of learning

In the specialized literature, it is possible to identify different models of SRL with empirical evidence that are organized or agglomerated into two large groups. On the one hand, there are those models based on social cognitive theory (Boekaerts and Niemivirta, 2000; Pintrich, 2000; Zimmerman, 2000) that are characterized by being strongly rooted in the self-efficacy framework (Bandura, 1991), and that assumes that people’s own beliefs in their efficacy contribute substantially to the various subprocesses in self-regulation (e.g., goal setting, self-monitoring, and the interpretation of causal attributions for success and failure; de la Fuente et al., 2022). In particular, two of these models emphasize motivational beliefs within the planning phase (Pintrich, 2000; Zimmerman, 2000). On the other hand, there are those models that are supported by cognitive and metacognitive aspects (Winne and Hadwin, 1998; Efklides, 2011), which are characterized to a greater extent by strategies referring to attentional control, monitoring, and evaluations of progress toward task goals. Although these models do not exclude motivational beliefs as relevant aspects, they emphasize mainly cognitive mechanisms (critical thinking, and problem-solving skills).

While there are a variety of SRL models with their own particularities or emphasis on the skills they include, it is certain that there is consensus regarding the following characteristics: (a) SRL can be developed, (b) self-regulatory behavior is the result of internal processes, including affective, cognitive, metacognitive, and motivational, (c) in general, three cyclical sequential phases are identified: Disposition, performance, and self-evaluation; (d) delineating these sequential phases allows understanding the behavior and use of different strategies that students exhibit in the pursuit of desired learning goals to achieve their purposes (Pogorskiy and Beckmann, 2023).

**Figure 1:**
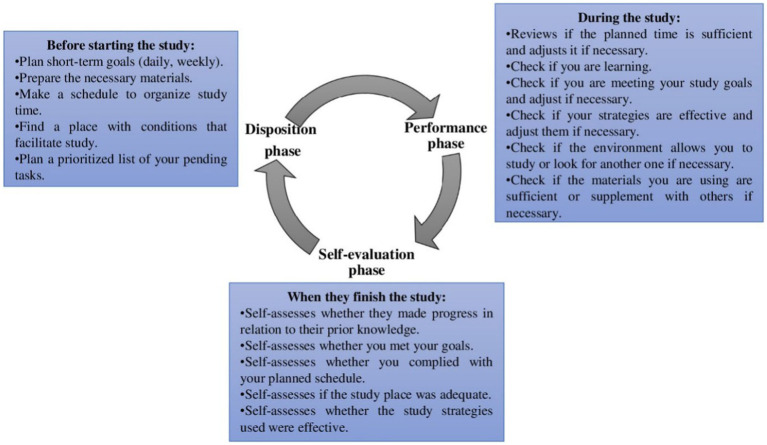
Specific strategies according to the phases of the empirical-theoretical model of self-regulation (Sáez-Delgado et al., 2022).

Therefore, this study is based on the proposal of a model that understands SRL as a cyclical process that takes place before, during, and after learning (see Figure 1). Specifically, before learning (“disposition phase”), learners analyze the task, set goals, and elaborate a specific plan to achieve the demands, all of these strategies are activated by motivational beliefs. During learning (performance phase), includes a variety of strategies that the learner uses for successful task completion when motivation is sufficient, such as, for example, monitoring planning, progress in meeting goals, a sufficient environment and materials for study, and whether these strategies are being effective or adjusting them if necessary. Finally, after learning (“self-evaluation phase”), where, after students complete their performance in coherence with the chosen objectives, they evaluate and react to their behaviors and performance results to attribute the possible factors that caused their success or failure (Sáez-Delgado et al., 2022). The product of this final phase of the SRL cycle impacts students’ motivational beliefs in future performance in which similar academic demands and requirements exist (Pogorskiy and Beckmann, 2023).

From the background, it is possible to define SRL as the proactive and diligent participation of students within a contextualized, dynamic and cyclical process, where they initiate, manage and adapt strategies for the pursuit of established objectives (see Figure 1), through which they can demonstrate the control of their own learning (Sáez-Delgado et al., 2022).

Therefore, a self-regulated student actively supervises and controls their learning, monitors the effectiveness of cognitive and metacognitive strategies in their study process, makes decisions to modify these strategies if necessary, so that they can achieve their goals, and show a high level of autonomy and determination in their learning to meet academic demands.

### 1.3. Reality of self-regulatory processes in secondary and higher education

Self-regulated learning is essential to ensure lifelong and productive learning in different contexts (Lim et al., 2023; Mustapha et al., 2023), and it is fundamental for students to persevere and succeed in their studies (Xu et al., 2023). Moreover, evidence abounds showing its positive association with academic outcomes such as grades (Dignath and Büttner, 2008; Pardo et al., 2017; Theobald, 2021; Lim et al., 2023), also, its association with non-academic outcomes (student satisfaction, student engagement and attitude toward learning), which are essential for learning progression in a university (Anthonysamy et al., 2020a), and, in general, for the benefits it brings toward efficient human capital for the future workplace. The value of SRL has even been demonstrated in complex, unprecedented, and conducive scenarios of deregulation such as the impact of the covid-19 pandemic on the education system (Holzer et al., 2021).

In the context of Higher Education, university students are expected to manage a lot of information and tasks in a more autonomous environment than they may have been used to in secondary school (Sáez et al., 2018; Sáez-Delgado et al., 2020). They often need to juggle simultaneously the high load of demands in the different courses, with their social life, in addition, in many cases the dedication to a part-time job (Jeong and Feldon, 2023). However, in Higher Education, university students often have insufficient strategies to regulate their own learning independently, suggesting the need for adequate support (Sáez-Delgado et al., 2020; Lobos et al., 2021; Jeong and Feldon, 2023; Lim et al., 2023). In fact, research has documented that, most frequently, students do not spontaneously regulate their learning (Lim et al., 2023), and lack sufficient knowledge and strategies to effectively complete and achieve their academic challenges, which highlights the lack of regulation in their study processes (Jeong and Feldon, 2023).

In the case of Secondary Education, the panorama is similar, and it is not strange to identify studies that affirm that students do not achieve optimal development of self-regulation strategies (Sáez-Delgado et al., 2021). Researchers have evidenced low use of planning strategies, difficulties to complete tasks or to monitor progress and solve problems (Lawanto et al., 2013), low self-efficacy (Bai and Wang, 2021), external causal attributions as responsible for their performances, especially in the case of students at risk of dropping out (Ardura et al., 2021), that is, they are more prone to attribute failure to uncontrollable factors than to controllable factors (Ngunu et al., 2019). This becomes complex, as the transition stage from secondary school to college can be particularly difficult for students, they need to be prepared to adapt to the independent learning environment of college, however, they are often insufficiently prepared to take responsibility for their own learning (Higgins et al., 2021; López-Angulo et al., 2023).

Although it is recognized that the ability to distinguish effective study strategies is associated with study planning that favors learning and that in addition, at the beginning of the career it is a determinant for the transit of students from secondary education to tertiary education, there does not seem to exist sufficiently solid bridges of self-regulatory skills that allow supporting the adjustment to university (Vosniadou, 2020; Blackmore et al., 2021). Because of the above, different efforts have been developed in universities to support students in the development of self-regulatory strategies for their study (Sáez et al., 2018). Although, a positive effect of these initiatives is shown, many times they are remedial and may be implemented late. For this reason, the focus is on Secondary Education, and studying the level of self-regulation at this educational level prior to university is of great importance (Ben-Eliyahu and Linnenbrink-Garcia, 2015; Sáez-Delgado et al., 2022).

SRL trajectories are likely to be substantially heterogeneous due to the dynamic and multidimensional nature of the self-regulatory process. Indeed, numerous cross-sectional studies have identified distinct profiles among students based on their use of SRL strategies (Dörrenbächer and Perels, 2016; Li et al., 2020; Sulisworo et al., 2020), suggesting the need for a longitudinal, person-oriented analytic approach to report heterogeneous SRL development (Jeong and Feldon, 2023).

Although the importance of understanding students’ self-regulatory processes for providing quality education is recognized, little is known about how students’ SRL profiles develop over time (Li et al., 2020), or the potential increase in students’ SRL at different grades along their academic trajectory, where they are supposed to be gaining educational experience (Higgins et al., 2021). The lack of a predefined and formal SRL trajectory makes it more difficult to assess students’ progress (e.g., by comparing it to a specific SRL baseline) and to provide relevant feedback and scaffolding, when appropriate (Mella-Norambuena et al., 2021).

### 1.4. Knowledge gap and aims of the current study

Given that the literature reveals that university students show significant study difficulties, and that the efforts of higher education institutions seem insufficient to reverse unsatisfactory experiences that, in many cases, lead to academic failure or dropout (López-Angulo et al., 2023), it is essential to focus on the level of development of self-regulation of learning in the pre-university stage (Sáez-Delgado et al., 2021, 2022). Currently, the results are limited in terms of the cross-sectional nature of the data, and therefore, authors have recently suggested the need to carry out research with a longitudinal design to confirm the self-regulation trajectories of students in secondary education (Lluch and Cano, 2023; Xu et al., 2023). It has even been suggested to analyze self-regulation trajectories considering the different grades of secondary education (Karademir and Deveci, 2019). This would make it possible to answer the knowledge gap regarding the possible variation in self-regulation levels as one progresses through different grades of secondary education (Jeong and Feldon, 2023). It would also facilitate understanding the current preparation of students to face university studies when they graduate from secondary education.

On the other hand, given the sex differences in secondary school students previously found in the literature on self-regulation (Torrano and Soria, 2017), it is considered important to include this variable since, if these differences are confirmed, this would provide valuable information for the design of special programs and training that attempt to develop self-regulation strategies. That is, it would be possible to suggest differentiated intervention modalities with emphasis on those self-regulatory processes that show more weaknesses in both men and women (Wu and Cheng, 2019).

Therefore, the present study implemented a longitudinal design and set as its general aim to evaluate the trajectories of self-regulation of learning (disposition, performance, and self-evaluation phase) in secondary school students during one academic year. The specific questions of the study are:

*RQ1*. Are there differences in self-regulated learning levels according to sex and grade?

*RQ2*. Are there variations in the levels of self-regulation of learning considering the interaction of the time variable and sex variable?

## 2. Materials and methods

### 2.1. Participants

For this study, two data collections on the same sample were considered. The first data collection (T1), was composed of a sample of 598 students, of whom 309 (51.7%) were female, 279 (46.7%) were male and 10 (1.6%) preferred not to state their sex. The mean age was 15.47 (SD = 1.16) years. Regarding the school level of the sample, 153 (25.6%) were 9th graders, 229 (38.3%) were 10th graders, 162 (27.1%) were 11th graders, and 54 (9.0%) were 12th graders. This sample was used to answer RQ1. The second data collection (T2), which was intended to follow up on the initial sample (T1), succeeded in obtaining responses from 403 students who participated in T1. This consisted of 202 males (50.1%) and 201 females (49.9%). In relation to school level, the sample consisted of 85 (21.1%) 9th grade students, 157 (38.9%) 10th grade students, 116 (28.8%) 11th grade students and 45 (11.2%) 12th grade students. All participants were Chilean students from secondary schools in the Biobío region of Chile.

### 2.2. Instruments

The Self-Regulation of Learning Instrument for Secondary Education Students (SRLI-SE) was used to measure the variable self-regulation of learning. The original and complete version of 34 items was validated for Chilean secondary school students (Sáez-Delgado et al., 2021). In this research, the abbreviated version of this instrument, previously used in Chile, was applied, showing adequate psychometric properties (Sáez-Delgado et al., 2022). Specifically, this instrument measures the learning self-regulation process by means of three scales in correspondence with the three phases proposed in the student self-regulation model (see Figure 1). The name of the first scale is “Disposition learning scale,” it has 5 items and measures the frequency with which students use self-regulation strategies to prepare their study, an example of items is: “Before I start studying, I plan short-term goals.” The name of the second scale is “Learning performance scale,” it has 6 items and measures the frequency with which students use strategies to control their study based on a previously established planning, an example of items is: “While studying, I check if I am learning.” Finally, the name of the third scale is: “Self-evaluation learning scale,” it has 5 items and measures the frequency with which students reflect on the results obtained in some task or school test, an example of items is: “When I finish my study, I self-evaluate if I made progress in relation to my previous knowledge.” The internal consistency of the three scales has shown to be adequate (Disposition: α > 0.79 and Ω > 0.82; Performance: α > 0.87 and Ω > 0.91; Self-evaluation: α > 0.85 and Ω > 0.87). The response format for each of the scales is the same, 7-point Likert-type (1 = never; 2 = almost never; 3 = seldom; 4 = half the time; 5 = frequently; 6 = almost always; 7 = always). Additionally, in this study, following previous examples from the literature (Verstege et al., 2019; Li et al., 2020; Jeong and Feldon, 2023) and in addition to validation by an expert panel composed of 5 PhDs with high expertise in the variable self-regulation and psychometrics, three SRL profiles were established. A longitudinal grouping approach of SRL level among learners determined by their frequency of use of self-regulation strategies was used: (a) Learners at optimal SRL levels (6–7 points), (b) Learners at suboptimal SRL levels (3–5), (c) Learners at insufficient SRL levels (1–2).

The questionnaire also included sociodemographic questions. Specifically, we asked about sex (the response options were: male, female, prefer not to answer); grade (the response options were 9th, 10th, 11th, and 12th) and the age variable.

### 2.3. Data collection procedure

To implement the study, it was first submitted for evaluation by the Institutional Ethics and Bioethics Committee of the Universidad Católica de la Santísima Concepción, Chile. Once the project underwent a detailed review and was approved by the Committee, meetings were arranged with secondary school principals to explain the research and invite them to participate. Those who agreed to participate in the research facilitated a meeting with the management team to agree on the data collection strategy, which included the prior authorization of the parents of the participating students, who signed an informed consent form, while the students approved their participation in the study through an informed consent form.

The data were collected at two moments over time to respond to the objectives of the study corresponding to the first and second academic semester of the year 2022 in Chile. Specifically, the first and second data collection was carried out from April to June and later from August to October, respectively. The instruments were applied online using the Surveymonkey tool. For the participants of this study, activities were carried out for the benefit of their schools. Specifically, a report was delivered with the overall results of the study and an invitation to a seminar where the findings were disseminated by the research team.

### 2.4. Data analysis procedure

In the first part of the study, which considered the evaluation of the variables at T1, descriptive frequency analyses were performed for the categorical variables. For numerical variables, central tendency and dispersion analyses were performed. Then, for the evaluation of differences in the variables of disposition, performance, and self-evaluation according to sex and educational level (grade), the assumptions of normality and homoscedasticity were evaluated using the Kolmogorov–Smirnov test with the Lilliefors modification and the Levene test, respectively. The results showed that both assumptions were not met and the groups were not balanced, therefore, it was decided to apply robust tests, for comparison by sex. Specifically, Yuen’s test was used for the comparison by sex, while for the comparison by educational level (grade), the trimmed means Anova test was used. Both tests are available in the WRS2 library.

In the second part of this study, where it was proposed to evaluate the effect of the interaction of time and sex on the variables disposition, performance, and self-evaluation, linear mixed models were used. The fixed effects considered were (1) evaluation time (T1 and T2), (2) sex (male or female), and (3) the interaction effect between evaluation time and sex. As a random effect, only the school intercept was considered in the model. To fit the linear mixed-effects model, the “lmer” function of the Lme4 library was used. The “ranova” function from the lmerTest library was used to evaluate the significance of the random effect in the model. All analyses were performed in R software version 4.2.2.2, with the RStudio IDE version 2023.03.0.

## 3. Results

### 3.1. Descriptive analysis of the variables

First, a descriptive analysis of the variable age and the SRL phases is presented. Table 1 shows that the variable disposition toward learning presented the highest mean *M* = 4.71; on the contrary, the variable with the lowest mean was the phase of self-evaluation of learning *M* = 4.25 (see Table 1). According to the interpretation of the instrument applied, it is possible to observe that, at T1, the learners show suboptimal levels of ARA.

**Table 1:** Descriptive analysis of the SRL phases.

	Mean	SD	Median	Skew	Kurtosis
Age	15.51	1.13	15.00	1.58	10.35
Disposition phase	4.71	1.43	4.80	−0.40	−0.25
Performance phase	4.61	1.50	4.67	−0.36	−0.45
Self-evaluation phase	4.25	1.59	4.20	−0.08	−0.75

### 3.2. Results of RQ1. Differences in SRL phases according to grade level and sex

#### 3.2.1. Results of the analysis in the SRL phases in the comparison by grade level

Differences by school level (grade) of the students were evaluated. The assumption of normality was not met and there was unbalance in the data, therefore, the robust trimmed means Anova test was performed. The analysis showed that there were no significant differences by level for the variables disposition, performance, and self-evaluation (see Table 2). According to the interpretation of the instrument applied, it is possible to point out that in T1 and in the different grades, the learner evidences suboptimal levels of ARA.

**Table 2:** Comparison analysis in the SRL phases according to grade level.

	9th grade (*n* = 153)		10th grade (*n* = 229)		11th grade (*n* = 162)		12th grade (*n* = 54)			
	Mean	SD	Mean	SD	Mean	SD	Mean	SD	Levene Test	ANOVA trimmed
DP	4.66	1.38	4.68	1.49	4.73	1.39	4.83	1.44	*F*(3,594) = 0.53	*F*(3,227.79) = 0.38
PP	4.58	1.44	4.56	1.58	4.68	1.47	4.75	1.43	F(3,594) = 0.96	*F*(3,227.45) = 0.54
SP	4.25	1.50	4.21	1.69	4.27	1.56	4.36	1.55	F(3,594) = 1.38	*F*(3,231.94) = 0.90

DP, Disposition Phase; PP, Performance Phase, SP, Self-evaluation Phase.

#### 3.2.2. Results of the analysis in the SRL phases in the comparison by sex

The sample for the comparison by sex was composed of 588 students. Students who preferred not to indicate their sex were eliminated from this comparison due to their low representativeness. The average age reported in this subsample was 15.46 (SD = 1.08) years. Regarding the level of study, 149(25.3%) were 9th graders, 226(38.4%) were 10th graders, 311(27.6%) were 11th graders, and 51(8.7%) were 12th graders.

Due to the failure to meet the assumptions for a two-group comparison with a parametric test, it was decided to use Yuen’s robust test. Significant differences according to sex were found for the variables disposition *T*(332.84) = 3.12. *p* < 0.01. ES 0.2 and for the variable performance *T*(352.66) = 1.97. *p* < 0.05. ES = 0.12 (see Table 3).

**Table 3:** Comparison analysis in the phases of SRL according to sex.

	Male (*n* = 279)			Female (*n* = 309)					
	M	SD	K-S Lilliefors	M	SD	K-S Lilliefors	Levene Test	Yuen-test	ES
DF	4.50	1.50	D = 0.051*	4.90	1.33	D = 0.057*	*F*(1,586) = 4.56*	*t*(332.84) = 3.12**	d = 0.2
PF	4.48	1.52	D = 0.056*	4.74	1.47	D = 0.063**	*F*(1,586) = 0.33	*t*(352.66) = 1.97*	d = 0.12
SE	4.19	1.62	D = 0.051*	4.30	1.56	D = 0.052*	*F*(1,586) = 0.22	*t*(346.87) = 0.64	N/A

DP, Disposition Phase; PP, Performance Phase, SP, Self-evaluation Phase.

### 3.3. Results of RQ2. Variations in the SRL phases considering the interaction of the time variable and the sex variable

#### 3.3.1. Descriptive results of the variables in the longitudinal study

Table 4 shows that the lowest mean of the variables studied was presented at T1 of the self-evaluation phase of the SRL process for the group of 12th grade secondary school males. On the other hand, the highest mean was presented at T1 of the disposition phase of the SRL process in the group of 12th grade secondary school females. At all times and phases of the self-regulation process, the group made up of women is the one with the highest averages. While, the lowest averages in all times and phases, is the one made up of men, except in the T2 self-evaluation phase.

**Table 4:** Description of the SRL phases according to sex, grade, and time.

			Edad	Disposition phase		Performance phase		Self-evaluation phase	
Sex	Grade	*n*	Mean	T1Mean (SD)	T2Mean (SD)	T1Mean (SD)	T2Mean (SD)	T1Mean (SD)	T2Mean (SD)
Male	9th	36	14.25	4.32 (1.59)	4.49 (1.77)	4.38 (1.58)	4.51 (1.79)	4.04 (1.68)	4.24 (1.81)
Male	10th	89	15.26	4.47 (1.69)	4.57 (1.6)	4.44 (1.7)	4.49 (1.59)	4.14 (1.8)	4.37 (1.64)
Male	11th	59	16.42	4.65 (1.39)	4.79 (1.46)	4.66 (1.47)	4.72 (1.49)	4.37 (1.5)	4.49 (1.51)
Male	12th	18	17.39	4.66 (1.3)	4.36 (1.38)	4.56 (1.49)	4.52 (1.17)	3.96 (1.61)	4.58 (1.28)
Female	9th	49	14.41	4.96 (1.31)	4.96 (1.6)	4.83 (1.38)	4.72 (1.74)	4.48 (1.47)	4.33 (1.8)
Female	10th	68	15.13	4.98 (1.3)	4.46 (1.4)	4.8 (1.52)	4.54 (1.54)	4.24 (1.59)	4.14 (1.63)
Female	11th	57	16.09	4.79 (1.46)	4.73 (1.46)	4.73 (1.57)	4.64 (1.57)	4.27 (1.69)	4.41 (1.61)
Female	12th	27	17.07	5.15 (1.54)	4.9 (1.42)	4.93 (1.53)	4.84 (1.59)	4.64 (1.64)	4.66 (1.46)

To evaluate the variation over time between measures of the variables of interest (SRL disposition phase, performance, and self-evaluation) and to test whether there is an interaction effect between time and sex, a linear mixed model was tested with time and sex as fixed effects and school as a random effect for the intercept.

#### 3.3.2. Linear mixed model on the SRL disposition phase

**Figure 2:**
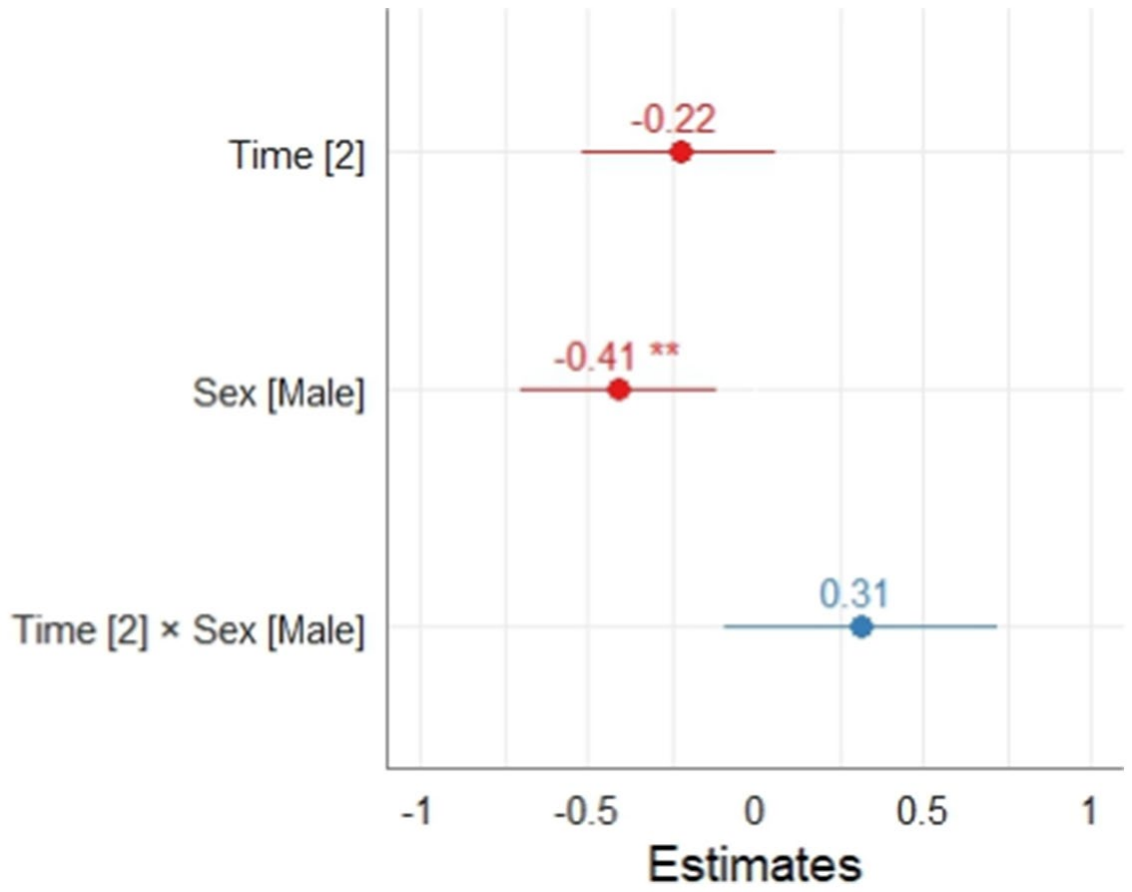
Effect of time and sex on the disposition phase of SRL.

The mixed model was evaluated considering the random effect of school only on the intercept. In this model it could be observed that the fixed effect of sex was significant (*p* < 0.01) in favor of females. On the other hand, time and the interaction between time and sex were not significant. According to the interpretation of the applied instrument, it is possible to point out that at T1 and T2 the students did not significantly change their level of disposition in the SRL process. Regarding the calculation of the conditional and marginal coefficient of determination for mixed-effects models, the results indicated an R2m = 0.010 and an R2c = 0.035. The calculation of the significance of the random effect of the school was significant *p* < 0.001 (Ver Table 5; Figure 2).

**Table 5:** Effect of time and sex on the disposition phase of SRL.

Predictors	Estimates	CI	*p*
(Intercept)	4.59	4.34–4.84	< 0.001
Time [2]	0.09	−0.20 – 0.38	0.543
Sex [female]	0.41	0.12–0.70	0.006
Time [2]* sex [female]	−0.31	−0.72–0.09	0.131
*Random effects*			
σ^2^	2.17		
τ_00school_	0.06		
ICC	0.02		
N_school_	19		
Observations	806		
Marginal *R*^2^/Conditional *R*^2^	0.010 / 0.035		

#### 3.3.3. Linear mixed model on SRL performance phase

**Figure 3:**
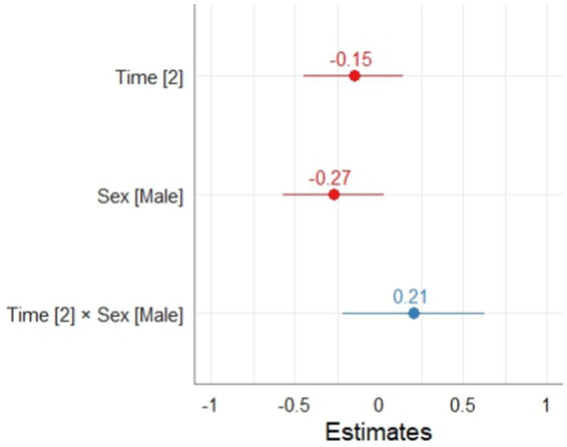
Effect of time and sex on the SRL performance phase.

In relation to the model for the learning performance phase in the SRL process, it can be observed that the fixed effect of sex, time and the interaction between them did not result significant. According to the interpretation of the applied instrument, it is possible to point out at T1 and T2 the students did not significantly change their level of performance of the SRL process. Regarding the calculation of the conditional and marginal coefficient of determination for mixed effects models, the results indicated an R2m = 0.004 and an R2c = 0.039, the calculation of the significance of the random effect was significant *p* < 0.001 (Ver Table 6; Figure 3).

**Table 6:** Effect of time and sex on the performance phase of SRL.

Predictors	Estimates	CI	*p*
(Intercept)	4.60	4.32–4.87	< 0.001
Time [2]	0.06	−0.24 – 0.36	0.704
Sex [female]	0.27	−0.03 – 0.57	0.080
Time [2]* sex [female]	−0.21	−0.63 – 0.21	0.334
*Random effects*			
σ^2^	2.33		
τ_00school_	0.08		
ICC	0.03		
N_school_	19		
Observations	806		
Marginal *R*^2^/Conditional *R*^2^	0.004/0.039		

#### 3.3.4. Mixed linear model on the SRL self-evaluation phase

**Figure 4:**
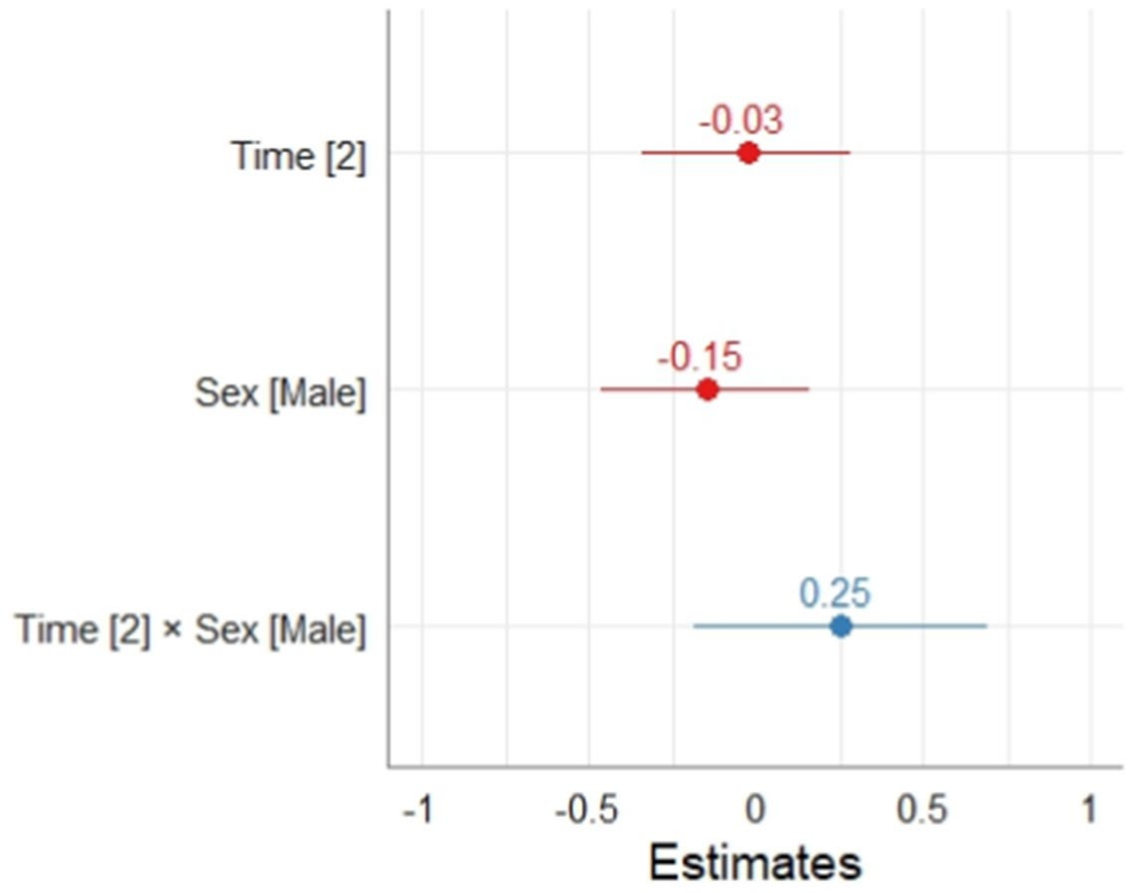
Effect of time and sex on the SRL self-assessment phase.

In relation to the model for the self-evaluation phase of learning, it can be observed that the fixed effect of sex, time, and the interaction between them did not result significant. According to the interpretation of the applied instrument, it is possible to point out that at T1 and T2 the students did not significantly vary their level of self-evaluation of the SRL process. Regarding the calculation of the coefficient of conditional and marginal determination for mixed effects models, the results indicated an R2m = 0.003 and an R2c = 0.055, the calculation of the significance of the random effect was significant *p* < 0.001 (Ver Table 7; Figure 4).

**Table 7:** Effect of time and sex on the self-evaluation phase of SRL.

Predictors	Estimates	CI	*p*
(Intercept)	4.30	3.99–4.60	< 0.001
Time [2]	0.23	−0.08 – 0.54	0.150
Sex [female]	0.15	−0.16 – 0.46	0.348
Time [2]* sex [female]	−0.25	−0.69 – 0.18	0.253
*Random effects*			
σ^2^	2.50		
τ_00school_	0.14		
ICC	0.05		
N_school_	19		
Observations	806		
Marginal *R*^2^/Conditional *R*^2^	0.003/0.055		

## 4. Discussion

This study aimed to answer two research questions that will be discussed below. Some reflections on the findings of this study and their implications for the quality of education, the limitations of the study, and future lines of research are also presented.

### 4.1. Discussion of RQ1. Differences in the levels of SRL according to sex and grade

Regarding the differences according to sex, the results showed significant differences in favor of females for the disposition phase and for the performance phase in the SRL process. This coincides with previous research and at different academic levels. In primary school a study involving 291 students from Hong Kong found sex differences in the use of SRL strategies, specifically in the strategies of planning, acting on feedback, and self-initiation in academic writing contexts in favor of girls (Bai et al., 2020). In secondary schools, similar results have also been found, for example, research with students from Turkey (Karademir and Deveci, 2019) and other studies with students from China in both face-to-face (Chen et al., 2023) and online learning contexts (Liu et al., 2021) found that self-regulation skills differ significantly as a function of sex in favor of female students. In Higher Education, these differences are also confirmed, for example, a study of 153 undergraduate students of Biological Education in Indonesia showed that female students are more self-regulated, specifically in goal setting, task strategies, time management, and self-evaluation (Anazifa et al., 2023); on the other hand, in a study in 700 undergraduate students of the Faculty of Foreign Languages in Turkey, similar results are revealed showing that female students are more self-regulated in comparison to male students (Adıgüzel and Orhan, 2017). Therefore, it is possible to conclude that the sex variable is a factor that differentiates students’ self-regulation skills in favor of female students at the school and university levels. This is further reinforced in research that has found that male students procrastinate in their studies more and show more addiction to smartphones than female students as a product of poor time management, variables directly associated with self-regulatory processes (Halili and Zainuddin, 2015).

Regarding differences by grade, data analysis did not detect significant differences in any of the self-regulation phases (disposition, performance, and self-evaluation). This is consistent with previous results in secondary education. For example, research on students in Turkey showed that self-regulation skills did not differ significantly as a function of grade (Karademir and Deveci, 2019). The same was concluded by a study that analyzed differences in 1,113 students in grades 11 and 12 of a public secondary school in China (Chen et al., 2023). On the other hand, an interesting result from a cross-sectional study of 1.260 Shanghai secondary school students in China (grades 10–12) analyzed whether the SRL level of these students varied across grades, although, these differences were not significant, still, the authors conclude from their findings of descriptive analyses that, in general, students’ SRL decreased as they got older (Guo, 2020); based on the analyses of mean differences by grade in the use of self-regulation strategies, since it was evidenced that 10th and 11th-grade students reported higher means compared to 12th-grade students in the strategies of rehearsal, elaboration, organization, metacognitive strategies, and intrinsic motivation.

### 4.2. Discussion of RQ2. Variations in the levels of SRL considering the interaction of the time variable and the sex variable

In the linear mixed models, significant differences by sex were found in favor of females for the learning disposition variable. This confirms the findings of the first objective cross-sectional cohort, which revealed that women are more self-regulated than men. However, in this case, differences are observed in the first phase of the cyclical SRL model, which is particularly important, since it is the phase that sets the self-regulation process in action. Also, other longitudinal studies have shown differences at the beginning of secondary school as a function of sex, with girls reporting greater use of self-regulation strategies compared to boys (Schuitema et al., 2012).

On the other hand, mixed-model results also indicated that the dispositional phases, performance, and self-evaluation of the SRL process do not change over time, i.e., students who responded regarding the frequency of self-regulation strategy use in one academic semester, and then those same students in a following academic semester, did not increase their SRL. This suggests that, while SRL strategies are not trained, these skills are not developed. These findings are also consistent with previous research results that have used longitudinal designs (Barbosa et al., 2018).

Other longitudinal studies have even shown more discouraging results. A longitudinal study on a sample of 412 Italian students between 12 and 22 years of age showed that self-regulatory self-efficacy decreased as they progressed through the following grades, with the decrease being greater and significant for males; specifically, self-regulatory efficacy decreased by 0.077 for males and 0.035 for females each year. In addition, a growth curve model showed that the smaller the decline in self-regulatory efficacy in the students’ trajectories, the higher the grades at the end of secondary school and the greater the probability of remaining in secondary school. The model explained 55% of the variance of dropout in males and 57% in females (Caprara et al., 2008). Another longitudinal study of 182 university students analyzed procrastination, which is considered a failure of self-regulation, measuring this variable at 4 moments during an academic semester, where the results showed that procrastination increased significantly throughout the semester (Yerdelen et al., 2016). A study on 735 students in the first year of lower secondary school in the Netherlands applied four measurements over time (the first in September/October 2004, i.e., at the beginning of the first year; the second in February/March; the third in May/June; and the fourth in September/October 2005, i.e., at the beginning of the second year). The results showed a relatively low use of strategies by the students in the different measures of the study where the authors point out that it could perhaps be due to the fact that the research participants were just starting to attend secondary school, where they become more responsible for their learning than in primary school. On the other hand, the results showed a general decrease in students’ perception of their self-regulated learning behavior as time progressed (van der Veen and Peetsma, 2009). Additionally, with respect to trajectories of self-regulation according to course, a study in 648 Netherlands secondary school students observed a decline in the first semester except for the use of metacognitive strategies, which remained the same [the SRL measure was measured at the beginning of secondary education and again in the middle of the first year (Schuitema et al., 2012)].

Therefore, it is possible to conclude from the findings of this study and on the support found in previous research, that secondary school students do not spontaneously improve their SRL by the mere fact of advancing from one academic semester to another. There seems to be stagnation and even regression of self-regulatory competence. It is possible to discuss some possible explanations regarding the lack of increased use of self-regulatory strategies during secondary school. First, is to consider the evolutionary perspective, i.e., as students move to the next semester or higher grade (increase in age), they acquire a greater ability to assess their actual rather than exaggerated competence, as opposed to when they were younger (van der Veen and Peetsma, 2009; Guo, 2020). Second, another possible explanation might find meaning in the underpinning of social cognitive theory (Bandura, 1999), which has emphasized that students might be influenced by their social environment (learning environment or school climate), i.e., while early adolescence is often characterized by a growing need for autonomy and self-awareness, the environment of the upper grades of secondary education becomes more evaluative, with more instances of formality and with a more competitive and impersonal character, implying a progressive undermining of SRL during this academic stage of students (Guo, 2020). This need for competition responds to the different demands that adolescents perceive in adult life (Pascoe et al., 2020). Thirdly, one should analyze the institutional emphasis that educational centers place on the development of competencies for life with different objectives, which are visualized and concretized in their mission and vision. In this sense, there are two big and interesting ideas when analyzing the purpose of education, one is related to preparing people to be productive and efficient in life and the other has to do with preparing them to manage their happiness and well-being (Alam, 2022). Considering that the Sustainable Development Goals (SDGs) and the 2030 Agenda propose to emphasize quality education, health, and well-being it is necessary to consider the development of transversal skills for self-management in life (English and Carlsen, 2019; Webb et al., 2019). At this point, it becomes imperative and necessary to reflect on how to move toward the transformation of education to play a leading role that contributes to resilient and sustainable happiness and development of well-being for all, this would reflect an innovative perspective that reinvigorates education and shapes the learning priorities of the 21st century (Malik, 2018), and therefore it would be necessary to consider the development of cognitive and emotional regulation to consolidate progress toward the challenge of quality in education (Parinussa et al., 2023).

In summary, whatever possible explanation is discussed regarding SRL levels in secondary school, it can be concluded that SRL does not increase as a natural consequence of human development; rather, it is learned and cultivated intentionally. Furthermore, from the results of this study and previous research, it is also concluded that SRL levels are lower than expected in order to adequately adjust to college.

### 4.3. Reflections on SRL and quality of education

The promotion of SRL in the school environment becomes relevant because it is associated with better academic experiences and outcomes, as well as with the general well-being of students (Rodríguez et al., 2022; Sverdlik et al., 2022). From this perspective, SRL can contribute to the reduction of existing gaps by equipping students with a key competency for their successful academic performance, thus increasing the possibilities of access to opportunities and professional academic training, which consequently favors the quality of education. This is supported by accumulated evidence, which has shown that beyond the sociodemographic variables which may describe situations of vulnerability in schools, those students who are self-regulated, achieve control of their study and learning process, advancing with determination toward the achievement of their personal and academic goals (Sáez-Delgado et al., 2021, 2022).

Therefore, the findings of this research on SRL in secondary school students contribute to the challenge promoted by the 2030 Agenda for Sustainable Development (English and Carlsen, 2019; Webb et al., 2019).That is, they are a valuable input for reflection and the elaboration of proposals that contribute positively to the academic formation of SRL strategies in secondary school students in Chile, this will allow ensuring a subsequent transition and successful adjustment to Higher Education. It is necessary to intend the development of self-regulatory competence of students in the pre-university stage, given the challenging contexts of academic demands typical of tertiary education where students must potentially face heavier academic workloads, practices or processes of stricter teaching-learning and with less guidance or supervision by teachers (Liu et al., 2021). Thus, the development of the ARA in secondary education will facilitate permanence or retention, avoid failure and academic dropout, especially in the first academic semesters at the university.

### 4.4. Limitations of the study and future lines of research

The results of this study need to be put in context and consider some limitations for their generalization. The first limitation of this study comes from the measurement of SRL, which consists of a self-report instrument, which could reveal some biases in the responses of the students who participated in the study, due to social desirability (Bensch et al., 2019; Vésteinsdóttir et al., 2019). Another limitation is regarding the data collection dates, corresponding to the first and second semesters of 2022, which coincide with the first academic year in Chile of return to face-to-face classes after physical isolation as a result of the COVID-19 pandemic. Therefore, it is necessary to consider that students could have a decrease in their SRL skills due to their previous learning experience in emergency remote teaching modality.

Regarding future lines of research, it is necessary that the SRL is intentionally pursued in the pre-university stage, that is, in secondary education. Ideally, it should become a specific learning outcome integrated into the subjects, to ensure that everyone who has passed this educational level goes on to higher education with a level of competence that facilitates their proper transition to a new academic context full of new challenges. For this reason, a study with a quasi-experimental design that is implemented in secondary education grades would be necessary to obtain a deep understanding of how SRL develops through interventions that push students toward the adoption of different self-regulation strategies to achieve their study goals (Lluch and Cano, 2023). An important challenge would also be to be able to define an expected sequence of different levels of SRL that is developed and demonstrated as they progress through the different grades of secondary education, thus promoting progressively greater autonomy and control of their learning process in students (Lluch and Cano, 2023). This would respond to a knowledge gap regarding how students’ SRL profiles develop over time in both secondary education and university (Li et al., 2020). The authorities of secondary schools are encouraged to consider among their strategies to advance toward the quality of education, the promotion of SRL in their centers (Obianujo, 2023). This promotion of ARA is a complex, dynamic and non-linear process that is likely to continue throughout the school and academic years, and should not be focused on a single experience. Therefore, the adoption of a perspective that is adapted to the multifactorial and long-term nature of self-regulatory capacities is required. Having a solid strategy to facilitate ARA in secondary education would avoid the development of compensatory or remedial interventions in the first years of university that may not be effective, thus, ecological interventions would contribute to a sustainable and quality education, leading to experiences and more successful academic trajectories that allow avoiding phenomena such as failure or dropout (Mustapha et al., 2023; Pogorskiy and Beckmann, 2023).

## Data availability statement

The original contributions presented in the study are included in the article/supplementary material, further inquiries can be directed to the corresponding author.

## Ethics statement

The studies involving human participants were reviewed and approved by Comité Ético Científico de la Universidad Católica de la Santísima Concepción, Concepción, Chile. Written informed consent to participate in this study was provided by the participants’ legal guardian/next of kin.

## Author contributions

FS-D contributed to the literature, abstract, and full-text review, as well as the data extraction, the data analysis, and the writing of the manuscript. JM-N contributed to well as the data extraction, the data analysis of the study, the interpretation of the results, and the writing of the manuscript. YL-A contributed to the design of the study, abstract, full-text review, interpretation of the results, and the writing of the manuscript. VL-R and YS contributed to the interpretation of the results, the writing of the manuscript, and full-text review. All authors contributed to the article and approved the submitted version.

## Funding

This study was funded by: FONDECYT Initiation Project N°11201054 entitled “The reciprocal relationship between teacher self-regulation and student self-regulation of learning and academic performance. An explanatory model in secondary education” of the National Research and Development Agency of Chile (ANID) assigned to FS-D.

## Conflict of interest

The authors declare that the research was conducted in the absence of any commercial or financial relationships that could be construed as a potential conflict of interest.

## Publisher’s note

All claims expressed in this article are solely those of the authors and do not necessarily represent those of their affiliated organizations, or those of the publisher, the editors and the reviewers. Any product that may be evaluated in this article, or claim that may be made by its manufacturer, is not guaranteed or endorsed by the publisher.
